# The cannabinoid receptor 1 associates with NMDA receptors to produce glutamatergic hypofunction: implications in psychosis and schizophrenia

**DOI:** 10.3389/fphar.2013.00169

**Published:** 2014-01-02

**Authors:** Pilar Sánchez-Blázquez, María Rodríguez-Muñoz, Javier Garzón

**Affiliations:** Neurofarmacología, Instituto Cajal, Consejo Superior de Investigaciones CientificasMadrid, Spain

**Keywords:** cannabinoid receptors, *N*-methyl-D-aspartate receptor, HINT1 protein, glutamatergic hypofunction, cannabis abuse, schizophrenia, psychosis vulnerability, G-protein-coupled receptors

## Abstract

The endocannabinoid system is widespread throughout the central nervous system and its type 1 receptor (CB1) plays a crucial role in preventing the neurotoxicity caused by activation of glutamate *N*-methyl-D-aspartate receptors (NMDARs). Indeed, it is the activity of NMDARs themselves that provides the demands on the endogenous cannabinoids in order to control their calcium currents. Therefore, a physiological role of this system is to maintain NMDAR activity within safe limits, thereby protecting neural cells from excitotoxicity. Thus, cannabinoids may be able to control NMDAR overactivation-related neural dysfunctions; however, the major obstacles to the therapeutic utilization of these compounds are their psychotropic effects and negative influence on cognitive performance. Studies in humans have indicated that abuse of smoked cannabis can promote psychosis and even circumstantially precipitate symptoms of schizophrenia, although the latter appears to require a prior vulnerability in the individual. It is possible that cannabinoids provoke psychosis/schizophrenia reflecting a mechanism common to neuroprotection: the reduction of NMDAR activity. Cannabinoids are proposed to produce such effect by reducing the pre-synaptic release of glutamate or interfering with post-synaptic NMDAR-regulated signaling pathways. The efficacy of such control requires the endocannabinoid system to apply its negative influence in a manner that is proportional to the strength of NMDAR signaling. Thus, cannabinoids acting at the wrong time or exerting an inappropriate influence on their receptors may cause NMDAR hypofunction. The purpose of the present review is to draw the attention of the reader to the newly described functional and physical CB1–NMDAR association, which may elucidate the scenario required for the rapid and efficacious control of NMDAR activity. Whether alterations in these mechanisms may increase NMDAR hypofunction leading to vulnerability to schizophrenia will be outlined.

## INTRODUCTION

Schizophrenia is a debilitating psychiatric illness that affects approximately 1% of the world’s population. The main symptoms associated with schizophrenia are grouped into three major symptom clusters: positive symptoms, negative symptoms, and cognitive disturbances (Lewis and Lieberman, 2000). Positive symptoms include visual and auditory hallucinations, delusions, and thought disorders. Negative symptoms include social withdrawal and anhedonia. Cognitive impairments are characterized by disturbances in sensory information processing, attention, working memory, and executive functions. The neural dysfunctions that underlie schizophrenia are likely the result of diverse genetic alterations acting in conjunction with environmental factors (Broome et al., 2005). The function of different neurotransmitter systems is altered in psychosis, such as that of dopamine, glutamate, gamma-aminobutyric acid (GABA), and adenosine. The systematic analysis of protein and gene expression suggested a possible convergence of these alterations on the glutamatergic system, particularly on the function of the *N*-methyl-D-aspartate (NMDA)-type ionotropic receptor, which mediates specific symptoms and features of schizophrenia (Mechri et al., 2001; Kristiansen et al., 2007; Moghaddam and Javitt, 2012).

In the central nervous system (CNS), the NMDA receptor (NMDAR) enables the permeation of calcium into the post-synaptic cleft and regulates essential processes, such as synaptic plasticity, learning, memory formation, and cognition. As a result, any dysfunction of this receptor in associative areas of the cortex can lead to alterations such as those observed in schizophrenia. NMDAR hypofunction is currently believed to provoke the dopaminergic deregulation observed in the striatal and prefrontal regions of schizophrenic patients (Harrison and Weinberger, 2005; Javitt, 2007), and that both of these abnormalities are believed to underlie the symptoms recognized as schizophrenia (Mohn et al., 1999). The most compelling evidence that supports the NMDAR hypofunction hypothesis comes from the effects of the NMDA antagonist phencyclidine (PCP). PCP, also known as “angel dust,” was found to induce psychotomimetic effects in healthy individuals that are similar to the positive, negative, and cognitive symptoms of schizophrenia, and it caused a resurgence of these symptoms in stable patients. PCP also targets acetylcholine and dopamine D2 receptors, however, its psychotomimetic effects are produced at serum concentrations that are selective for the NMDAR (revised in Javitt and Zukin, 1991). Therefore, nervous tissue development anomalies or a series of genetic alterations promoting NMDA receptor hypofunction may lead to the development of schizophrenia symptoms. According to this idea, enhancers of NMDAR currents have the potential to ameliorate the symptoms of schizophrenia, while antagonist-mediated impairment of NMDAR’s activity produces psychotic symptoms and others schizophrenia-associated symptoms (Abi-Saab et al., 1998).

The endocannabinoid system controls the activity of NMDARs, preventing their overactivation and providing neuroprotection of neuronal cells against excitotoxicity. Cannabinoid-mediated control of NMDAR function has been observed in functional studies (Thorat and Bhargava, 1994; Derkinderen et al., 2003; Marsicano et al., 2003; Liu et al., 2009), in hippocampal slice cultures (Khaspekov et al., 2004), and in cultured neurons (Kim et al., 2006). The excitotoxicity mediated by experimental activation of NMDARs is enhanced in cortical cultured neurons following targeted deletion of the cannabinoid receptor type 1 gene (*CNR1*; Kim et al., 2006; Vicente-Sánchez et al., 2013). Since in this paradigm alternative systems do not appear to exert significant control, CB1 emerges as a decisive negative physiological regulator of NMDAR function in the earliest development of cerebral cortical structures. Several studies have indicated that cannabinoids oppose glutamatergic NMDAR function through various mechanisms, such as the pre-synaptic reduction of glutamate release into the cleft (Brown et al., 2003; Melis et al., 2004; Li et al., 2010), or the inhibition of post-synaptic cannabinoid receptors whose signaling pathways may interfere with those of NMDARs (Liu et al., 2009; Hampson et al., 2011). Another possibility is that cannabinoids prevent endogenous calcium increases via mechanisms associated with the direct inhibition of the NMDAR channel (Zhuang et al., 2005; Liu et al., 2009; Li et al., 2010).

Thus, exogenous cannabinoids acting at the wrong time or exerting an inappropriate influence on the endocannabinoid system may cause a disproportionate downregulation of NMDAR activity (hypofunction; Javitt and Zukin, 1991; Javitt, 2007). This possibility is consistent with the reported relationship between cannabis use and schizophrenia, particularly as it relates to the amount of cannabis used in adolescence and the subsequent risk of developing schizophrenia, or the appearance of schizophrenia-like symptoms in non-schizophrenic people after cannabis use (Fernandez-Espejo et al., 2009). Further, biological phenomena related to endogenous cannabinoids, such as significantly higher amounts of the endocannabinoid anandamide in the blood and cerebrospinal fluid occur more frequently in patients with acute schizophrenia than in healthy volunteers (Leweke et al., 1999; De Marchi et al., 2003). While cannabis use in the general population does not affect the incidence of schizophrenia it does reduce the age of onset of psychotic illness among vulnerable individuals, in whom the endogenous cannabinoid system would otherwise promote the onset of this condition at a slower rate (Degenhardt et al., 2003).

We now present an overview of the functional relationship between CB1 and NMDARs, outlining the influence that their recently described association in the brain may play in these mood disorders. This novel information may provide more precise clues to schizophrenia vulnerability when CB1-mediated regulation of NMDAR function fails and produces glutamatergic hypofunction.

## CANNABINOIDS AND SCHIZOPHRENIA

The pharmacological administration of endocannabinoids, as well as synthetic and exocannabinoids, induces a full range of transient positive, negative, and cognitive symptoms in healthy individuals that are similar to those observed in schizophrenia (Fernandez-Espejo et al., 2009). However, only a small subset of the population of smoked cannabis abusers develops psychotic illness. Cannabis exposure is actually believed to be a “component cause” that interacts with other decisive factors to precipitate schizophrenia and other psychotic disorders, but is neither necessary nor sufficient to do so alone. Despite a series of data supporting the hypothesis that certain genetic alterations contribute to cannabis-mediated precipitation of schizophrenia, conflicting evidence exists regarding the hierarchy of these polymorphisms and how they relate to the endocannabinoid or glutamatergic systems. Therefore, different genetic and pathophysiologic mechanisms have been hypothesized to underlie different forms of schizophrenia (Leroy et al., 2001; Chavarria-Siles et al., 2008). As our knowledge increases, these mechanisms may converge.

Endocannabinoids transmit their signals through two well-characterized cell surface receptors, CB1 and CB2, which belong to the superfamily of G-protein-coupled receptors (GPCR). Functional evidence suggests the existence of other receptor subtypes, although their identities have been elusive so far. CB1 is primarily expressed in neural tissue and is also present in peripheral tissues. This receptor is likely the most abundant of all known GPCRs, with densities 10- to 50-fold above those of classical neurotransmitters, such as dopamine or opioid receptors (Howlett et al., 1990; Herkenham et al., 1991). CB2 is mostly expressed by immune cells in peripheral tissues and is present to a lesser extent in various regions of adult rat and mouse brains (Van Sickle et al., 2005; Gong et al., 2006). Within cortical areas, both receptors show notable differences in their neuronal distribution, which may indicate that they have non-overlapping functional roles. Therefore, both cannabinoid receptors are believed to regulate important physiological activities in the CNS by working independently and/or cooperatively in different neuronal populations (Onaivi et al., 2006).

CB2 may be altered in schizophrenia, and clinical remission of schizophrenia is accompanied by significant decreases in anandamide and CB2 mRNA levels in peripheral blood mononuclear cells (De Marchi et al., 2003). However, it is not clear whether such changes are also produced by cannabinoid treatment in healthy individuals. Interestingly, targeted deletion of the *CNR2* gene causes schizophrenia-related behaviors in mice (Ortega-Alvaro et al., 2011). This genetic finding is also supported by the results of pharmacologic experiments that were conducted in a mouse model of schizophrenia; the CB2 antagonist AM630 enhanced MK801- and methamphetamine-induced disturbances of the prepulse inhibition (PPI; Ishiguro et al., 2010). The authors concluded that reductions in CB2 function alone do not cause schizophrenia, but may be harmful for schizophrenia-susceptible individuals. Similarly, CB2 agonists reduced stereotypic behaviors that are related to schizophrenia-associated hyperdopaminergic functioning (Onaivi et al., 2006). These results are in agreement with the observation that CB2 agonists provide neuroprotection against 6-hydroxydopamine toxicity *in vivo* and *in vitro* (Garcia-Arencibia et al., 2007). Evidence of the direct functional or molecular relationship between CB2s and NMDARs has not been provided in the literature. The protective effects of CB2 activation against glutamate-induced excitotoxicity are mediated through the activation of microglia and the release of endogenous IL-1ra (Molina-Holgado et al., 2003). However, the CB1 subtype can establish direct interactions with NMDARs (Sánchez-Blázquez et al., 2013b; Vicente-Sánchez et al., 2013) and it mediates the majority of the neuroprotective effects of cannabinoids (Marsicano et al., 2002; Derkinderen et al., 2003; Liu et al., 2009). It is therefore probable that CB2 hypofunction causes alterations in signaling pathways directly and negatively coupled to NMDAR function, of which CB1 is a candidate.

The CB1 regulates both pertussis toxin-sensitive Gi/o proteins and pertussis toxin-insensitive Gz proteins (Garzón et al., 2009), and its distribution in nervous tissue is consistent with the effects of cannabinoids on emotional responses, cognition, memory, movement, and nociception (Herkenham et al., 1991; Howlett, 1995; Walker and Huang, 2002). The activation of CB1 specifically can produce long-lasting functional changes in the glutamatergic system, and prenatal exposure of rats to CB1 agonists causes a series of alterations in cortical NMDAR signaling that affect cognition (Antonelli et al., 2005). Moreover, repeated exposure to Δ9-tetrahydrocannabinol (Δ9-THC) impairs hippocampal long-term potentiation (LTP) of excitatory glutamatergic transmission and diminishes the expression of NMDARs (Fan et al., 2010).

Given the essential role of NMDAR dysfunction in psychotic illnesses, for the purposes of this review, we will focus our attention upon the CB1. The constraints that CB1 activity imposes on NMDAR function may underlie both neuroprotection and the negative effects of cannabinoids, and thus smoked cannabis abuse may lead to psychosis and circumstantially precipitate or intensify symptoms of schizophrenia (Degenhardt et al., 2003; Fernandez-Espejo et al., 2009), albeit mostly in subjects bearing a previous vulnerability (Cannon and Clarke, 2005;Harrison and Weinberger, 2005). Considering that CB1 activity is recruited on-demand to control NMDAR signaling, the efficacy of this regulation may rely on the proportionality of both activities; and dysregulation of this process could certainly lead to persistent glutamate NMDAR hypofunction. The relationship between dopamine receptors and NMDARs is complex, and while NMDAR calcium fluxes increase in response to activated D1 and D2 receptors, the D4 receptor reduces NMDAR function (Beaulieu and Gainetdinov, 2011). NMDARs can form regulatory complexes with dopamine D1 receptors and likely with D2 receptors (Fiorentini et al., 2003; Pei et al., 2004). The NMDARs activated by D1/D2 receptors could be recruited to negatively control dopamine signaling, similar to the effect they have on mu-opioid receptors (MOR; Rodríguez-Muñoz et al., 2012; Sánchez-Blázquez et al., 2013a). The glutamatergic pathway projecting from cortical pyramidal neurons to the ventral tegmental area controls dopaminergic neurons via the activity of GABA interneurons. In this regulatory neural circuit NMDAR hypofunction causes dopamine hyperfunction of the mesolimbic dopamine pathway (Gaspar et al., 2009). If NMDARs and D1/D2 receptors establish such regulatory associations, then NMDAR hypofunction would increase dopamine activity contributing to the symptoms of schizophrenia.

## A NEW PERSPECTIVE: THE ASSOCIATION OF CB1s WITH NMDARs

A series of biochemical, molecular, and pharmacological studies have demonstrated the functional interaction between the MOR and the CB1 (Desroches and Beaulieu, 2010), as well as the interaction of these GPCRs with the glutamate NMDAR (Rodríguez-Muñoz et al., 2012; Sánchez-Blázquez et al., 2013b). NMDARs are primarily targeted to the post-synaptic region of glutamatergic synapses, where they are organized (and spatially restricted) into large macromolecular signaling complexes that contain scaffolding and adaptor proteins. In these structures, NMDARs physically link to kinases, phosphatases, GPCRs, and other signaling molecules (Kim and Sheng, 2004; Sans et al., 2005). Immunocytochemical and ultrastructural studies have described the presence of CB1s in the post-synapse at both the spinal (Hohmann et al., 1999; Ong and Mackie, 1999; Salio et al., 2002) and supraspinal levels (Rodriguez et al., 2001; Kofalvi et al., 2005), where they co-localize with NMDARs (Marchalant et al., 2008). Co-immunoprecipitation assays performed on mouse cerebral cortical synaptosomes have also demonstrated the association between CB1 and NMDARs, concretely with NR1 subunits (Garzón et al., 2009; Sánchez-Blázquez et al., 2013b). In these assays, PSD95 proteins co-precipitated with CB1 confirming the presence of this cannabinoid receptor at the post-synapse. The second PDZ domain in PSD95 binds to the C-terminal amino acid sequence that is common to NR2 subunits and NR1 C2′ splice variants (Kornau et al., 1995) enabling CB1 to co-precipitate the PSD95 via NR1 subunits (Sánchez-Blázquez et al., 2013b). Pull-down and surface plasmon resonance studies that were performed using recombinant proteins revealed the direct physical interaction between the C-terminal region of CB1 and the C1 segment of the NMDAR NR1 subunit. The direct interactions between these proteins were also detected in a normal cellular environment using the bimolecular fluorescence complementation (BiFC) assay (Shyu et al., 2008). Chinese hamster ovary (CHO) cells were transfected with a mixture (1:1) of plasmids expressing CB1 coupled to VC155 and NR1 (C0-C1-C2) coupled to VN173 at the corresponding C-termini. The physical interaction of these carrier proteins allows the VC155 and VN173 fragments to couple and form a stable fluorescent complex, and numerous cells displayed a fluorescent signal indicating that CB1 and NR1 interact to form heterodimers *in vivo* (Sánchez-Blázquez et al., 2013b).

**FIGURE 1:**
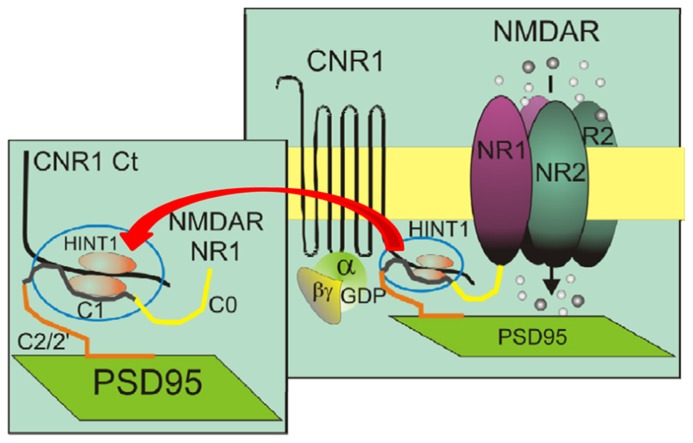
**The role of HINT1 proteins in the direct interaction between the C-terminal sequences of CB1 and the NMDAR NR1 subunits.** Both CB1 and the NR1 subunits interact with the HINT1 protein (Sánchez-Blázquez et al., 2013b). This model describes the physical interaction between the CB1 C-terminus and the NR1 C1 segment, as well as both proteins with the HINT1 protein, which strengthens their association.

The dopamine D1 receptor (Fiorentini et al., 2003), the mGlu5a metabotropic glutamate receptor (Perroy et al., 2008), and the MOR (Rodríguez-Muñoz et al., 2012) are all examples of GPCR–NMDAR operative complexes. Histidine triad nucleotide-binding protein 1 (HINT1) associates with the C terminus of MOR and CB1 (Guang et al., 2004; Sánchez-Blázquez et al., 2012), where it behaves as a scaffold protein that brings together a series of signaling proteins (Rodríguez-Muñoz and Garzón, 2013). The HINT1 protein appears to be essential for the ability of these GPCRs to regulate NMDARs (Rodríguez-Muñoz et al., 2011; Sánchez-Blázquez et al., 2013b; Vicente-Sánchez et al., 2013). Its role in sustaining this regulatory connection will also be discussed. The potential of the recently reported association between CB1s and NMDARs to influence cannabinoid-induced psychosis was suggested by a series of studies on NMDARs and CB1-induced analgesia. Pretreatment with NMDAR non-competitive antagonists, such as MK801, greatly reduced the capacity of cannabinoids to produce analgesia (Palazzo et al., 2001; Sánchez-Blázquez et al., 2013b). These *in vivo* observations suggested that NMDAR activity was required for cannabinoids to promote antinociception (Urca et al., 1980; Jensen and Yaksh, 1989). However, while MK801 greatly reduces the analgesia displayed by WIN 55,212-2 in wild-type mice, it fails to do so in HINT1^-^^/^^-^ mice (Sánchez-Blázquez et al., 2013b). In these mice, CB1 is disconnected from the NMDAR and cannabinoids do not protect cultured cortical HINT1^-^^/^^-^ neurons from NMDA-induced excitotoxic insults (Vicente-Sánchez et al., 2013). Thus, cannabinoid-mediated analgesia does not require NMDAR activity. Further, *in vitro* and *ex vivo* studies revealed that the C-terminal sequences of the CB1 and of the NR1 subunit of the NMDAR can establish direct physical interactions and that the HINT1 protein stabilizes their association and makes it functionally relevant (**Figure 1**; Sánchez-Blázquez et al., 2013b; Vicente-Sánchez et al., 2013). In this framework, CB1 activation promotes the co-internalization of NMDAR NR1 subunits with little or no NR2 subunits. MK801 by binding simultaneously to NR1 and NR2 subunits in the NMDAR pore forming region strengthens their interactions (Kashiwagi et al., 2002), and CB1 then couples to the whole NMDAR. Cannabinoid production of potent supraspinal analgesia requires of CB1 internalization/recycling-resensitization, yet the MK801-bound NMDAR prevents cannabinoids from efficiently internalizing CB1s, which rapidly desensitize at the plasma membrane producing the described reductions in the analgesic effects of cannabinoids. As expected, in HINT1^-^^/^^-^ mice, MK801 fails to impair cannabinoid-induced internalization of CB1s. Thus, CB1–NMDAR complex does not contribute to cannabinoid-mediated antinociception, although it is required for endocannabinoids to negatively regulate the function of the ionotropic glutamate receptor (Sánchez-Blázquez et al., 2013b).

## FUNCTIONAL STUDIES: CANNABINOIDS RESTRICT NMDAR FUNCTION AND PROTECT NEURAL CELLS FROM EXCITOTOXIC INSULTS

Several studies have reported cannabinoid-mediated protection of cell viability in different models of neurotoxicity, such as that evoked by oxidative stress, the β-amyloid peptide, and focal cerebral ischemia. However, this protection may not always be mediated through pathways that are related to CB1 or CB2 activation (Marsicano et al., 2002; Harvey et al., 2012). In the present review, we will focus on neuroprotection derived from the negative control of NMDAR activity that natural, synthetic or endogenous cannabinoids produce via their binding with CB1s (Khaspekov et al., 2004; Liu et al., 2009; Sánchez-Blázquez et al., 2013b). The activity of NMDARs is positively regulated by a series of GPCRs via protein kinase C (PKC) and Src (Lu et al., 1999; Salter and Kalia, 2004). NMDAR overactivation typically produces an excess of calcium influx and subsequent calcium release from internal organelles. Such excessive activation augments the activity of NMDAR-coupled neuronal nitric oxide synthase (NOS) and NO then reacts with superoxides to form peroxynitrite, generating reactive oxygen species (ROS) and releasing zinc from intracellular stores upon oxidative and nitrosative stimulation. The latter metal ion contributes significantly to toxicity by damaging critical metabolic enzymes and contributing to the engagement of apoptotic cascades (Frederickson et al., 2005; Knott and Bossy-Wetzel, 2009). Zinc homeostasis seems particularly sensitive to oxidative stress and its dysregulation appears to enhance neurotoxicity, most likely because differences in zinc load activate distinct harmful pathways (Aizenman et al., 2000; Frederickson et al., 2004; Sensi and Jeng, 2004). The endocannabinoid system is probably the endogenous regulator that most effectively restrains NMDAR activity reducing calcium influx and the subsequent release of calcium and zinc from endogenous stores (Sensi and Jeng, 2004; Zhuang et al., 2005; Hampson et al., 2011; Sánchez-Blázquez et al., 2013b).

CB1 activation mobilizes a large number of signal transduction pathways, and the neuroprotective antioxidant effects of cannabinoids are also dependent upon the inhibition of Ca^2^^+^ influx through voltage-gated Ca^2^^+^ channels (Mackie and Hille, 1992) and the suppression of cyclic adenosine monophosphate (cAMP)-mediated protein kinase A (PKA) signaling (Kim et al., 2006). Cannabinoids primarily control the activity of exogenous activators of NMDARs by acting on the calcium channel, rather than stimulating calcium clearance by reuptake or its expulsion into the extracellular space (Sensi and Jeng, 2004; Zhuang et al., 2005). The post-synaptic CB1–NMDAR association may facilitate such endogenous control, although this mechanism appears to fail or is overridden by the activation of NMDARs under certain neuropathic circumstances, as well as in certain neurodegenerative illnesses.

## CB1–NMDAR ASSOCIATION AND GLUTAMATE HYPOFUNCTION

In addition to other interactions that may occur between the endocannabinoid and glutamate systems, the CB1–NMDAR association establishes a new scenario in which the CB1 directly interacts with the NMDAR to reduce its activity. A relevant aspect of this regulation is the fact that the endocannabinoid system is being recruited for NMDAR activation and that its response must be fast enough to achieve an efficacious restriction of NMDAR calcium permeation. Otherwise, as the interval between the activation of NMDARs and CB1 response increases this control is diminished (Zhuang et al., 2005). This is because NMDAR function recruits the activity of PKA, via Ca^2^^+^-CaM/adenylyl cyclase/cAMP, which displaces the negative influence of CB1 activation on NMDAR function (Sánchez-Blázquez et al., 2013b). Thus, an endocannabinoid system that is permissive of the glutamate activity could eventually cause excitotoxic stress-induced neural dysfunctions. Excessive cannabinoid-mediated restriction of NMDAR function may precipitate glutamate hypofunction such as that observed in schizophrenia. In regard to CB1–NMDAR association, cannabinoids that regulate NMDAR function promote the internalization/recycling of the cannabinoid receptor to reduce the stability of the glutamate receptor through the co-internalization of NR1 subunits (Garzón et al., 2009; Sánchez-Blázquez et al., 2013b). This mechanism disassembles the NMDAR and increases the presence of NR1 subunits in the cytosol, subsequently increasing the presence of NR2 subunits as well (Fan et al., 2010; Sánchez-Blázquez et al., 2013b).

**FIGURE 2:**
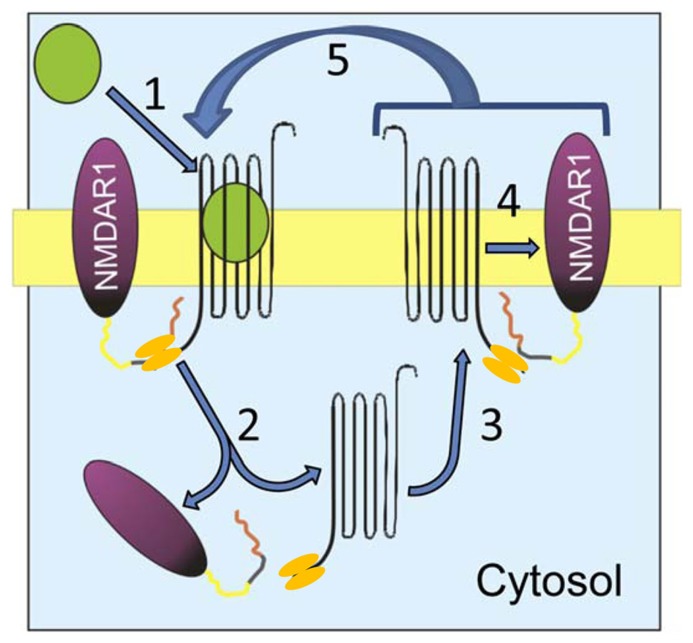
**Proposed mechanism of cannabinoid-induced NMDAR hypofunction.** CB1 and the NMDAR NR1 subunit associate in the post-synapse via the homodimeric HINT1 protein. (1) The agonist binds to CB1 and (2) promotes co-internalization of CB1–HINT1 and NR1 subunits. These proteins separate in the cytosol and (3) CB1–HINT1 returns to the plasma membrane. (4) The re-sensitized CB1 associates with new NR1 subunits and (5) the cycle is reinitiated while the agonist remains in the receptor environment.

Precise knowledge of the molecular mechanisms that enable this physiological regulation will permit the identification of the signaling elements that cause NMDAR dysregulation. In the cell membrane, the physical association of CB1–NMDAR and its functional relevance are dependent upon the HINT1 protein (Sánchez-Blázquez et al., 2013b; Vicente-Sánchez et al., 2013). Following agonist challenge, CB1 is co-internalized with HINT1 and NR1 subunits, and whereas the HINT1 protein conserves its association with the CB1, it dissociates from the NR1 subunits. When the CB1–HINT1 complexes are recycled back to the cell surface, they rapidly re-associate with new NR1 subunits for their co-internalization. As the agonist-internalized CB1s return to the cell surface within a few minutes (Hsieh et al., 1999), the presence of exogenous cannabinoids in the receptor environment produces rapid and repeated recycling of CB1s, which may cause instability and the disruption of many NMDARs (**Figure 2**). Obviously, if exogenous cannabinoids drive this regulation beyond physiological limits, the number of NMDARs on the cell surface diminishes as they are progressively driven to lysosomes and metabolized, leading to glutamate hypofunction. However, it remains unclear as to whether a safe switch disconnects NMDAR function from the negative influence that the disproportionate activation of the CB1 produces in such circumstances.

In this context, the HINT1 protein represents a promising target that can be used to modulate cannabinoid neuroprotection. This protein has been found to be associated with the cytosolic regions of a variety of GPCRs (Sánchez-Blázquez et al., 2012), and molecular studies have indicated that this scaffold protein determines the quality of the GPCR–NMDAR interaction. CB1s and MORs associate via HINT1 proteins with NR1 subunits. Whereas MOR agonists disrupt the HINT1–NR1 association and trigger negative feedback via increases in NMDAR responsiveness, cannabinoids maintain the HINT1–NMDAR association, which is necessary to counteract excitotoxicity and preserve cell viability. These differences could account for neuropathies in which NMDAR hyperactivity diminishes the antinociceptive capacity of strong analgesics such as opioids, but cannabinoids still display some of their analgesic effects (Ashton and Milligan, 2008). Given the fact that several GPCRs engage functional cross-talk with ion channels, these interactions may underlie both the therapeutic and undesirable side-effects of currently available drugs.

## CONCLUSION AND PERSPECTIVE

The negative regulation of NMDARs by cannabinoids is particularly relevant because their persistent activation produces a series of perturbations that may lead to neurodegenerative diseases (Lipton, 2006), mood disorders, such as depression (Maeng and Zarate, 2007), and neuropathic pain (Sigtermans et al., 2009). The CB1–NMDAR association plays a significant role in the efficacy of cannabinoids in combating NMDAR hyperactivity-induced insults. In a demanding model, such as that of NMDA application-induced neuronal excitotoxicity, the agonists that promote strong CB1s internalization, such as WIN55,212-2 and ACEA (Garzón et al., 2009), provide more effective protection against neural injury than anandamide or methanandamide which barely internalize this receptor (Kim et al., 2006; Kreutz et al., 2007). Such a pattern is consistent with cannabinoids reducing the stability of the NMDAR, thereby dampening its activity.

As mentioned previously, schizophrenia is related to alterations that lead to NMDA hypofunction and dopaminergic hyperfunction in cortical/subcortical areas. Most relevant, neurobiological studies indicate that cannabis and endocannabinoids can dysregulate the neurotransmitter systems involved in the pathophysiology of schizophrenia, such as the dopaminergic and glutamatergic systems (Javitt, 2007). Indeed, cannabinoids reduce hippocampal LTP, which is related to NMDAR function, and have been implicated in learning and memory processes. Additionally, cannabinoid abuse produces dopaminergic hyperfunction in limbic areas and the cortex, which may cause the cannabinoid-induced cognitive deficits. This enhancement of dopamine function appears to be caused by CB1-mediated NMDAR hypofunction (Javitt, 2007). A relevant question is whether the association between CB1 and the NMDAR may provide clues as to the nature of susceptibilities implicated when smoked cannabis abuse promotes psychosis and circumstantially precipitates symptoms of schizophrenia (Harrison and Weinberger, 2005).

To produce effective control of NMDAR activity, the cannabinoid system must equilibrate its negative influence to the strength of NMDAR signaling (Marsicano et al., 2002). If this does not occur, the lack of harmonization may provoke NMDAR-mediated cell damage or the excessive downregulation of glutamate signaling, which may also negatively affect cell homeostasis. While the duration of such effects is limited and the system can be recovered and reset to normality, disproportionate CB1-mediated control of NMDAR activity may reduce its recovery and produce persistent NMDAR hypofunction. Therefore, a poor or excessive CB1-mediated effect on NMDAR activation may cause a series of neural dysfunctions in the long term. The extent of the endocannabinoid response is dependent upon the NMDAR-induced recruitment of CB1s and/or the mechanisms disconnecting both receptors to prevent NMDAR hypofunction. Alterations in the NR1 subunits, CB1 and/or the HINT1 protein on serine/threonine kinases implicated in CB1 recruitment to NR1 subunits may cause the vulnerability required for exogenous cannabinoids to precipitate psychosis and eventually schizophrenia.

**FIGURE 3:**
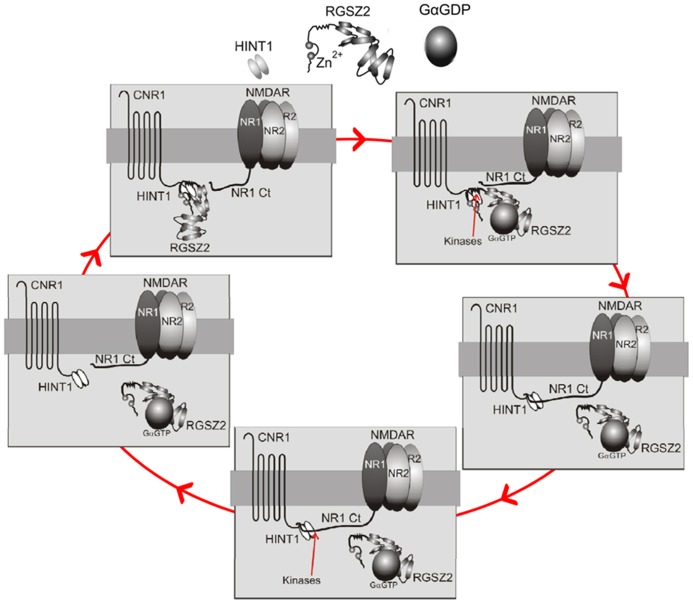
**Diagram describing the recruitment of CB1-mediated control of NMDAR activation.** *Upper left panel*: Under resting conditions the CB1–HINT1 complex remains bound to the RGSZ2 protein. *Upper right panel*: Upon activation of NMDARs, endogenous cannabinoids act on CB1s and activate G proteins. Some GαGTP subunits bind to the RGS domain of RGSZ2 proteins and expose HINT1 to kinases, such as CB1-activated PKC (Rodríguez-Muñoz et al., 2011). *Middle right panel*: This action forces the release of RGSZ2 and promotes the binding of the NMDAR NR1 subunit to the HINT1 protein (Sánchez-Blázquez et al., 2013b). *Lower panel*: The association of NR1s with CB1s permits cannabinoids to reduce NMDAR function, and the ratio between NMDAR activity and CB1 recruitment will be dependent upon the extent of NMDAR-mediated calcium fluxes (most likely by a calcium sensor), and the activity of kinases that favor the formation of this complex. *Middle left panel*: When NMDAR activity ceases the silent NMDARs will be protected by the return of RGSZ2 to the HINT1 protein (the action of other kinases on the NR1 subunit likely reduces its binding to HINT1, or alternatively, the action of phosphatases on HINT1 favors RGSZ2 binding). Note 1: In the absence of cannabinoid activity, there are probably numerous CB1–NMDAR complexes in which the NMDAR is active, but its function is limited (recall that in the absence of CB1s or HINT1 proteins, NMDAR activity increases; Kim et al., 2006; Sánchez-Blázquez et al., 2013b). The aforementioned mechanism may therefore affect the increase in NMDAR activity, which will reach a given threshold (these putative calcium sensors are essential for controlling this). The release of silent NMDARs from the control of activated CB1s is essential to avoid unnecessary glutamate hypofunction. Note 2: In the absence of CB1 activation, a large increase in NMDAR activity, such as that provided by intracerebroventricular (icv) injection of NMDA, forces the PKA-mediated separation of NMDARs from the CB1 and the loss of any potential control (see the transit from the lower panel to the upper left panel).

Our current knowledge indicates that the HINT1 protein is critical for this regulation. Indeed, in the absence of HINT1, the NMDAR cannot establish functional or molecular interactions with CB1, and cannabinoids fail to reduce NMDAR-mediated calcium flux, NO production, and calcium and zinc release from endogenous stores (Sánchez-Blázquez et al., 2013b). Thus, the HINT1 protein enables CB1–NMDAR cross-regulation and may also be decisive for the harmonization of their activities (**Figure 1**). CB1 couples via HINT1 proteins to a regulator of G protein signaling, the RGSZ2 protein (also named RGS17), which binds to neural NOS (nNOS) and controls NO production via CB1 activation (Marsicano et al., 2002; Garzón et al., 2011; Rodríguez-Muñoz and Garzón, 2013). One possibility is that NR1 subunits and RGSZ2–nNOS complexes compete for HINT1 proteins. Thus, upon simultaneous activation of CB1s and NMDARs, kinases, such as PKC or calmodulin-dependent protein kinase II (CaMKII) disrupt the HINT1/RGSZ2–nNOS association (Rodríguez-Muñoz et al., 2011), favoring HINT1 binding to NR1 subunits. In contrast, the release of NR1 subunits from the CB1 environment stimulates the association of RGSZ2 proteins with this receptor. This phenomenon may couple the formation of CB1–NMDAR complexes with the levels of NMDAR activity and uncouple CB1 from the source of NO (nNOS) and zinc ion production, the RGSZ2–nNOS complex (Garzón et al., 2011; Sánchez-Blázquez et al., 2012), thereby preventing cannabinoids from contributing NO/zinc to the process of glutamatergic excitotoxicity. The plasticity of this regulatory process may help adjust the CB1–NMDAR association to the level of NMDAR activation, making the control exerted by cannabinoids over NMDARs more efficient and importantly, protecting against unnecessary NMDAR hypofunction (**Figure 3**).

The physical and functional association of CB1s with NMDARs requires HINT1. Targeted deletion of the *HINT1* gene uncouples the NMDAR from CB1 inhibition and the activity of the glutamate receptor thus increases (Sánchez-Blázquez et al., 2013a). In the plasma membrane and in the context of CB1–NMDAR regulation, the HINT1 protein may behave as a circuit breaker in this CB1–NMDAR association, releasing NMDARs from the control of CB1 and enhancing their binding to RGSZ2–nNOS complexes (although only when the CB1–NMDAR association produces excessive NMDAR hypofunction). It is possible that this security mechanism is triggered in response to insufficient NMDAR permeation of calcium or calcium–calmodulin levels. Therefore, alterations in this mechanism which senses NMDAR function may promote NMDAR hypofunction. Indeed, the HINT1 protein has been related to psychosis (Vawter et al., 2002, 2004), and, the *HINT1* gene is located on chromosome 5q31.2, a region implicated in linkage studies of schizophrenia. Thus, an anomalous HINT1 protein or alterations of its endogenous regulators could contribute to cannabinoid-induced NMDAR hypofunction. The diminished expression of *HINT1* mRNA found in post-mortem brains of schizophrenia patients may reflect a compensatory mechanism leading to reduce the expression of such anomalous HINT1 proteins. Moreover, data from association and expression studies suggest that variants of *HINT1* may be associated with schizophrenia and that these associations may be sex-specific (Chen et al., 2008).

In summary, the existence of HINT1-dependent CB1–NMDAR associations provides a new interaction to consider when evaluating the capacity of cannabinoid abuse to produce NMDAR hypofunction, such as that observed in psychosis and schizophrenia. As *HINT1* has been described as a susceptibility gene for schizophrenia, variants of the HINT1 protein found in schizophrenic patients may help enhance the NMDAR hypofunction caused by exogenous cannabinoids that trigger the activation of the endogenous regulatory system at the wrong time, with excessive frequency and intensity. Anomalous increases in the affinity of the CB1 C-terminus for the C1 segment of the NR1 subunits or diminished HINT1–RGSZ2 interactions may cause a disproportionate disruption of NMDARs upon CB1 internalization, and the HINT1 protein emerges in this context as a potential therapeutic target. Accordingly, dysregulation of the molecular mechanisms responsible for CB1-mediated control of NMDAR activity may constitute risk factors that could precipitate symptoms of schizophrenia in early cannabis abusers that may otherwise be induced by the endogenous system, albeit at a later time.

## Conflict of Interest Statement

The authors declare that the research was conducted in the absence of any commercial or financial relationships that could be construed as a potential conflict of interest.
